# Health status of adolescents in the Tibetan plateau area of western China: 6 years after the Yushu earthquake

**DOI:** 10.1186/s12955-017-0727-4

**Published:** 2017-07-29

**Authors:** Xu Liu, Hongyang Yang, Bihan Tang, Yuan Liu, Lulu Zhang

**Affiliations:** 1 Department of HealthService, Faculty of HealthService, Second Military Medical University, 800Xiangyin Road, Shanghai, 200433 China; 2 Department of Medical Affairs, The General Hospital of the PLA Rocket Force, Beijing, China

**Keywords:** Health status, Earthquake, Child, Risk factor

## Abstract

**Background:**

An earthquake struck Yushu in Qinghai province of China on April 14, 2010, causing 2698 deaths and 12,135 injuries.The present study aimed to assess the health status, and associated determinants, of child survivors in the epicenter of the Yushu earthquake 6 years after the event.

**Methods:**

A cross-sectional survey was performed among students from two junior schools in Yushu County. Descriptive statistics, t-tests, ANOVA, Wilcoxon rank sum tests, Kruskal-Wallis H tests and stepwise linear regression analysis were used for data analysis.

**Results:**

The mean scores onmental component summary (MCS)and physical component summary (PCS) were 42.13 (SD 7.32) and 42.04 (SD 8.07), respectively. Lower PCS in the aftermath of an earthquake was associated with being trapped/in danger, injured to self, receiving no escape training while lowerMCS in the aftermath of an earthquake was associated with a lower grade level, not living with parents, fear during the earthquake, death in the family, and not receiving psychological counseling after the earthquake.

**Conclusions:**

In conclusion, the results of the present study help to expand our knowledge regarding the health status of child survivors 6 years after the Yushu earthquake. Our study provides evidence-based suggestions for specific long-term health interventions in such vulnerable populations.

## Background

Not only can natural disasters cause a devastating loss of life, homes, and livelihoods in an instant, they can also impose lingering threats to the physical and mental health of the survivors who must cope with post-disaster adversities for many years [1, 2]. Moreover, earthquakes are one of the most severe forms of natural disaster. Among developing countries, China is one of the most affected by natural disasters. A catastrophic earthquake, registering 7.1 on the Richter scale, struck Yushu, China, at 7:49 am on April 14, 2010, causing 2698 deaths and 12,135 injuries. The epicenter of the earthquake, Yushu, is a county in the Yushu Tibetan Autonomous Prefecture in the southernmost Qinghai province, with an average elevation of 4493 m. The total population of Yushu has been reported to be 311,000 (in 2007), of which Tibetans accounted for 97%, and the agricultural and animal husbandry population accounted for 70% [3].

There is an emerging literature on the health status of earthquake survivors [4–7], with some studies contending that severe earthquakes/disasters can cause long-lasting physical and mental health problems [8]. Previous studies have assessed the impact of disasters in terms of mortality, morbidity, depression, posttraumatic stress disorder (PTSD), and the health-related quality of life (HRQoL) within a 1–36-month period following the disaster [9–15]. Most of these studies have focused on adults; only a few have examined the health status of child survivors [16, 17]. Moreover, no previous study has evaluated the health status of child survivorsseveral years after an earthquake.

Child survivors are a vulnerable population whose health status is sensitive to external adverse events such as earthquakes. Several studies focusing on child survivors have shown that the HRQoLdecreases over time. Enabling children who have survived disasters to achieve and maintain the same standards of health as other children and adolescents is a goal of the international community [18].

In particular, for relatively remote plateau areas like Yushu, the health status of child and adolescent survivors should be continuously considered. Therefore, the present study aimed to assess the health status, and associated determinants, of child survivors in the epicenter of the Yushu earthquake(the undeveloped Tibetan plateau area, Northwest China) 6 years after the event, to provide evidence-based suggestions for specific long-term health interventions in such vulnerable populations.

## Methods

### Study design and participants

A cross-sectional survey was conducted in August 2016, at which time 6 years had passed since the event [19]. The capital of Yushu Autonomous Prefecture is Jiegu, which has 8 junior/middle schools and one vocational school. In China, mostjunior schoolshave3 grades (grades 1–3), which are equivalent to the grades 6–8in occidental education systems;senior grades 1–3 are similar to grades 9–12 in occidental education systems. Chinese junior school students are12–16 years of age. The local summer vacation in Yushu is May through June, 2 months earlier than most of China. May through June is also the harvest season for cordyceps (Yushu is the hometown of cordyceps) and the junior students provide the major labor force in their family in unearthing cordyceps. One of the 8 junior schools, Nang Qian No. 2 Middle School, was randomly selected for our study sample. In addition, we investigated the students from the vocational junior middle school, Limin Vocational Middle School. In contrast to Nang Qian No. 2 Middle School, the junior students educated at Limin Vocational Middle School typically work directly after graduation instead of going to a junior middle school for further study. The initial study sample was comprised of all junior students (grades 1–3) present at the time of our evaluation who had personally experienced the Yushu earthquake. A total of 709 junior students were approached; 103 were excluded as they did not personally experience the Yushu earthquake and 3 refused to participate in the investigation. Thus, a total of 603 junior students completed the questionnaire; however, 12 questionnaires were excluded due to missing items. Therefore, the total number of participants was 591.

### Data collection

The survey was conducted by 12 undergraduates from the Second Military Medical University. The Project Leader conducted a centralized training in Shanghai for the 12 investigators on August 12, 2016, briefed the prospective respondents about the details of the study, and answered questions related to the study. The 12 researchers were divided into 6 groups and were dispatched to Yushu County to conduct the investigation from the 13th to the 26th of August, 2016. The survey took place in the school classrooms. Given the limited comprehensive ability of the middle school students, each groupof researchers briefed the prospective respondents, providing details of the study, and answered related questions. Approval was obtained from the school principal who made an announcement regarding the study to the students. The study also obtained approval from the ethics committee of Second Military Medical University, and all participants voluntarily agreed to participate in our survey.

### Measurements

The collected social and demographic information included sex, age, ethnicity, religious faith, chronic disease history, residential area, family resident population, main source of income for the family, whether living with parents or not, whether an only child or not, and family type. Students “not living with parents” refer to the left-behind children (i.e., the biological parents wentto the city to work, leaving the child behind tostay with grandparents or other living relatives).

Earthquake-related experiences were assessed using an inventory adapted from a commonly used earthquake exposure scale. The items included ‘being trapped or in danger’, ‘house damaged’, ‘family property damaged’, ‘injured’, ‘family member injured’, ‘lost family member or friend’, degree of fear during the earthquake, degree of fear, etc. [20].

The health status of the students was assessed using the acute version of the Chinese version of the Short Form-12 (SF-12), which is a practical, reliable, and valid scale adapted from the 36-item Short Form health survey (SF-36). The Chinese version of the SF-12 has been validated for Chinese adolescents [21]. The participants were asked to complete the SF-12 by recalling their personal experience for the previous 4 weeks. The SF-12 has been used in numerous studies worldwide, and includes one item from each of the bodily pain, general health, vitality, and social functioning scales, and two items from each of the physical functioning, role-physical, role-emotional, and mental health scales of the SF-36 [22].In addition, the SF-12 assesses the overall physical and mental functioning using two summary scores: the physical component summary (PCS) score and mental component summary (MCS) score [23].The MCS and PCS scores were calculated usingthe methods proposed by Ware et al. [24] The SF-12 takes only two to 3 min to complete and higher scores reflect better health [6].

### Data analysis

All analyses were performed using the Statistical Package for the Social Sciences (SPSS) Version 11.0 (SPSS Inc., Chicago, USA). Descriptive statistics (frequencies, percentages, means, and standard deviations [SD]) were first calculated. Subsequently, t-tests (for two-group comparisons) and analyses of variance (ANOVAs; for multi-group comparisons) were used to evaluate differences in continuous variables when the data were normally distributed and in accordance with assumptions regarding the homogeneity of variance; otherwise, non-parametric methods were used (Wilcoxon rank sum tests for two-group comparisons and Kruskal-Wallis H tests for multi-group comparisons).For ANOVAs with a *p* values <0.05, multiple comparison tests were conducted using the least significant difference(LSD) test. A multiple linear regression including all the variables was used to find the factors independently associated with PCS and MCS scores [25]. Meanwhile, a stepwise linear regression analysis with 0.05 for entry and 0.1 for staying was performed to identify the predictors of PCS and MCS scores, and the beta coefficients (B), standardized error of the coefficient (SEB), and statistics are reported. The PCS and MCS scores of the Yushu adolescent survivors were compared to those of the general adolescent population in China using a one sample t-test [21]. A *p* < 0.05 was considered statistically significant.

## Results

### Social-demographic characteristics and earthquake-related experiences

Social-demographic characteristics and earthquake-related experiences are presented in Table 1. Of the 591 participants, 297 (50.25%) were male, and 459 (77.67%) were in Grades 1 or 2. The participants’ ages at the time of the survey ranged from 11 to 18 years, with an average age of 15.49 years. The vast majority of students (570, 96.45%) were of Zang descent (the major ethnic population in Yushu County) and 586 (99.15%) had a religious faith. In terms of residence, only 72 students (12.18%) lived in the downtown area while 207 (35.03%) lived in the country and 312 (52.79%) lived in the pasture. A total of 488 (82.57%) students lived with their parents. During the earthquake, 502 participants (84.94%) felt fearful; 426 (72.92%) reported that no family members were injured; and 325 (54.99%) reported that no family members were killed due to the earthquake. Furthermore, 484 (81.9%) participants were not personally injured, but 219 (37.06%) reported being in danger or trapped during the earthquake. Just under half of the participants (*n* = 252, 42.64%) reported that their houses had been damaged during the earthquake. Similarly, less than half of the students (*n* = 222, 37.56%) reported that their family experienced property loss due to the earthquake. A total of 318 participants (53.81%) had received psychological escape training and counseling.

**Table 1:** Demographics and earthquake-related experiences among adolescent survivors of the Yushu earthquake, China

Variable	N	Percentage (%)	PCS		MCS	
			Mean	*p*	Mean	*p*
Total	591	-	42.13(7.32)		42.04(8.07)	
Gender				0.6531		0.1602
Male	297	50.25	42.26(7.5)		42.5(8.12)	
Female	294	49.75	41.99(7.14)		41.57(8.01)	
Grade				0.0724		<0.0001*
1	202	34.18	42.8(7.36)		40.28(7.83)	
2	257	43.49	41.35(6.99)		42.2(8.12)	
3	132	22.34	42.63(7.78)		44.39(7.77)	
Religious belief				0.8200		0.9621
Yes	586	99.15	42.12(7.35)		42.03(8.08)	
No	5	0.85	42.67(2.44)		42.21(7.9)	
Residence				0.4415		0.4284
Downtown	72	12.18	42.46(7.79)		42.96(7.73)	
Country	207	35.03	41.6(7.83)		41.56(8.59)	
Pasture	312	52.79	42.4(6.85)		42.14(7.79)	
Living with parents				0.2408		0.0021
Yes	488	82.57	41.97(7.42)		42.5(7.81)	
No	103	17.43	42.9(6.82)		39.82(8.92)	
Trapped or in danger				<0.0001		0.9316
Yes	219	37.06	39.99(7.18)		42.07(8.1)	
No	372	62.94	43.39(7.12)		42.01(8.07)	
House damage due to earthquake				0.0314		0.3900
Yes	252	42.64	41.38(7.09)		42.3(7.93)	
No	339	57.36	42.69(7.44)		41.84(8.18)	
Property loss due to earthquake				0.1509		0.3900
Yes	222	37.56	41.57(7.42)		42.4(7.92)	
No	369	62.44	42.46(7.25)		41.81(8.16)	
Fear during the earthquake				0.0615		0.6183
No	40	6.77	41.91(8.29)		40.99(8.11)	
A little	110	18.61	42.88(7.23)		41.81(7.58)	
Much	127	21.49	42(7.99)		41.48(8.17)	
Extremely	265	44.84	41.46(6.91)		42.35(8.12)	
Unclear	49	8.29	44.58(6.6)		43.12(8.7)	
Injury to self due to earthquake				0.0002		0.5664
Yes	107	18.1	39.78(7.92)		42.44(8.7)	
No	484	81.9	42.65(7.08)		41.95(7.93)	
Injury in the family due to earthquake				0.0442		0.7103
Yes	165	27.92	41.16(7.02)		41.84(8.32)	
No	426	72.92	42.51(7.41)		42.11(7.98)	
Death in the family due to earthquake				0.5614		<0.0001
Yes	266	45.01	42.32(7.25)		40.4(7.66)	
No	325	54.99	41.97(7.38)		43.38(8.16)	
Escape training				0.0002		0.0563
Yes	318	53.81	43.17(7.03)		41.45(8.11)	
No	273	46.19	40.92(7.47)		42.72(7.99)	
Psychological counseling				0.9913		0.0320
Yes	318	53.81	42.13(7.43)		42.7(7.66)	
No	273	46.19	42.13(7.43)		41.27(8.47)	

* multiple comparison tests:Grade 1 versus Grade 2: *p* < 0.05; Grade 1 versus Grade 3: *p* < 0.05; Grade 2 versus Grade 3: *p* < 0.05

### Factors associated with the physical component summary score

The mean PCS score was 42.13 (SD 7.32),which was significantly lower than the PCS of the general adolescent population in China (42.13 vs. 49.8, *P* < 0.0001).In bivariate analyses (Table 1), PCS scores were negatively and significantly associated with the following factors: ‘being trapped or in danger’ (*p* < 0.001), ‘house damaged’ (*p* = 0.0314), ‘injured’ (*p* = 0.0002), ‘family member injured’ (*p* = 0.0442), and not receivingescape training (*p* = 0.0002). Multiple stepwise regression analyses (Table 2) revealed that PCS scores 6 years after the earthquake were negativelyassociated with ‘being trapped or in danger’ (*p* = 0.0001), ‘injured’ (*p* = 0.0323), and not receiving escape training before the earthquake (*p* < 0.0001).

**Table 2:** Multiple stepwise regression analysis of factors independently associated withPCS scores

Label	Initialmodel^a^		regression model^b^	
	β coefficients	*P value*	β coefficients	*P value*
Degree	-0.35036	0.4365		
Gender(Male(1), Female(2))	-0.70773	0.2667		
Religious belief(Yes (1),No (2))	2.72113	0.4034		
Residence ^c^				
Country	-1.44545	0.1602		
Pasture	-0.90759	0.3572		
Living with parents(Yes (1),No (2))	0.99805	0.2224		
Trapped or in danger(Yes (1),No (2))	2.75754	0.0005	2.5594	0.0001
House damage due to earthquake (Yes (1),No (2))	-0.13789	0.8549		
Property loss due to earthquake (Yes (1),No (2))	-0.53195	0.466		
Fear during the earthquake (No(1), A little(2),Much(3),Extremly(4),Unclear(5))	-0.19195	0.5319		
Injury to self due to earthquake (Yes (1),No (2))	2.14019	0.0159	1.7709	0.0323
Injury in the family due to earthquake (Yes (1),No (2))	0.39888	0.605		
Death in the family due to earthquake (Yes (1),No (2))	-0.5046	0.444		
Escape training(Yes (1),No (2))	-2.51614	0.0001	-2.5272	<0.0001
Psychological counseling (Yes (1),No (2))	0.40026	0.5262		

^a^ initial model included all variables listed in Table 1

^b^ The inclusion criteria of the stepwise regression was p = 0.05; exclusion criteria was p = 0.10

^c^ referencegroup=Downtown

### Factors associated with the mental component summary score

The mean MCS score was42.04 (SD 8.07), which was significantly lower than the MCS of the general adolescent population in China (42.04 vs. 45.4, *P* < 0.0001).In bivariate analyses (Table 1), MCS scores were negatively associated with death in the family (*p* < 0.0001) and were positively associated with a higher grade level (*p* < 0.0001), living with parents (*p* = 0.0021), and receiving psychological counseling (*p* = 0.0320). Multiple stepwise regression analyses (Table 3) revealed that lower MSC scores 6 years after the earthquake were associated with a lower grade level (*p* = 0.0006), not living with parents (*p* = 0.0084), fear during the earthquake (*p* = 0.0081), death in the family (*p* = 0.0005), and not receiving psychological counseling after the earthquake (*p* = 0.0275).

**Table 3:** Multiple stepwise regression analysis of factors independently associated with MCSscores

Label	Initialmodel^a^		regression model^b^	
	β coefficients	*P value*	β coefficients	*P value*
Degree	1.53198	0.0019		
Gender(Male(1), Female(2))	-0.13103	0.8501	1.6025	0.0006
Religious belief(Yes (1),No (2))	-1.24924	0.7246		
Residence ^c^				
Country	-0.47612	0.6708		
Pasture	0.11338	0.9159		
Living with parents(Yes (1),No (2))	-2.41344	0.0069	-2.3023	0.0084
Trapped or in danger(Yes (1),No (2))	-0.13443	0.875		
House damage due to earthquake (Yes (1),No (2))	-0.34196	0.6771		
Property loss due to earthquake (Yes (1),No (2))	-0.63483	0.4243		
Fear during the earthquake (No(1), A little(2),Much(3),Extremly(4),Unclear(5))	-0.91053	0.0066	-0.8737	0.0081
Injury to self due to earthquake (Yes (1),No (2))	-0.52918	0.583		
Injury in the family due to earthquake (Yes (1),No (2))	-0.72773	0.3864		
Death in the family due to earthquake (Yes (1),No (2))	2.69157	0.0002	2.3567	0.0005
Escape training(Yes (1),No (2))	0.73534	0.2979		
Psychological counseling (Yes (1),No (2))	-1.64898	0.0168	-1.4770	0.0275

^a^ initial model included all variables listed in Table 1

^b^ The inclusion criteria of the stepwise regression was p = 0.05; exclusion criteria was p = 0.10

^c^ referencegroup=Downtown

## Discussion

Experiencing a disastrous earthquake can result in long-term mental and physical impairment among adolescent survivors [17, 26].The present study examined the health status, both in terms of physical and mental health, of adolescent survivors 6 years after the Yushu earthquake. We found that adolescent survivors of the Yushu earthquake had worse PCS and MCS scores compared to those for the general adolescent population in China [21].

With regard to the physical health status of adolescent survivors, being trapped and injured during the earthquake were found to be risk factors with a strong negative association to physical health. While a large number of studies have focused on the mental health problems of survivors [27–30], research on the relationship between exposure to disaster events and survivors’ physical health is relatively limited. A previous study found that post-earthquake rescue policies could directly affect the promotion of PCS scores in survivors [31]. However, additional studies have suggested that disaster events have little impact on survivors’ physical health [27].For example, the results of the study by Adams and Boscarino suggested that exposure to the World Trade Center disaster did not have severe consequences for the physical well-being of adults 1 year after the attacks [32]. In other previous studies, poor housing conditions (such as living in temporary shelters and a lack of clean water and food) were reported to have a negative association with physical health 1-18 months after the disaster [6, 33, 34]. Our findings surprisingly show that even after a fairly long period of 6 years, being trapped and injured during the earthquake still had a strong negative association with physical health. These results may be related to dysfunction and movement disorders caused by the earthquake injury, such as amputations, becoming crippled, and so on. Overall, the results suggest that long-term follow-up for earthquake-related injuries and relevant measures should be developed to promote recovery.

In the present study, earthquake escape training was shown to have a positive association with physical health. Community emergency preparedness/engagement is critical for effective rescue, and earthquake escape training an important measure to enhance community emergency preparedness/engagement [35, 36]. Earthquake-related health education and promotion can bring positive changes to populations in areas at high risk for earthquakes. An improved level of knowledge and consequent preparedness are far more promising than the “treat when sick” strategy [37].

With regard to the psychological health status of adolescent survivors, being afraid and losing family members during the earthquake corresponded to lower MCS scores. These negative factors have been identified in previous cross-sectional studies with greater exposure to the events surrounding the disaster associated with poorer psychological well-being [5, 17]. Moreover, the results of the present study are consistent with previous studies demonstrating that children who felt intensely scared during a disaster were more likely to have poor psychological health status and were more likely to develop symptoms of PTSD, depression, and anxiety [38, 39]. In addition, our results indicated that although 6 years had passed, the students’ emotional memory of the Yushu earthquake was still clear. As suggested in previous studies, experiencing the loss of family members as a result of the earthquake may have aroused intense fear, which was closely related to negative psychological changes [40–43]. Since disaster-related psychological sequelae may last for many years, appropriate actions should be taken to minimize potential negative impacts. A regular, long-term psychological intervention program should be established to improve the mental health conditions of survivors in earthquake-affected areas.

On the other hand, we found that receiving psychological counseling was associated with higher MCS scores. This result provides a comforting message, suggesting that appropriate psychological interventions can be effective in improving the mental health of survivors. In the presentstudy, psychological counseling included counselingimmediately after the disaster or several days tomonths later, either in individual or group settings, and to any extent. A wide variety of studies have appealed for more attention to the mental health of survivors [44–46]. However, few studies exist on how to implement and assess the effectiveness of psychological interventions.

It is noteworthy that, in addition to the above earthquake exposure factors, two demographic characteristics also had a strong association with the MCS score: the adolescents’ grade level and whether they were living with their parents. A higher grade level and living with parents were positively associated with MCS scores. A previous study focusing on resilience reported the opposite result (i.e., junior grade 1 students hadnotably higher resilience scores compared to those of senior grade 1 students, which was attributed to senior grade 1 students facing great pressure to perform well in academics and meet the high expectations of parents) [47]. Since the population living in the Yushu area is scattered, the two investigated schools were boarding schools; thus, the students were usually living in the school. The lower grade students had just started the school’s collective life. Therefore, we speculate that the impact of grade level on the MCS score might be due to low-grade students not yet adapting to the school atmosphere and living situation. Regarding the association between living with parents and MCS scores, China has been undergoing rapid urbanization in the past few decades and one out of ten individuals is a migrant, moving from rural to urban areas to seek employment opportunities. Therefore, a considerable number of participants had left the rural area, and were not living with their parents. Evidence from studies in various countries suggests that a stable family environment contributes to the healthy development of children [48–53]. In addition, negative impacts such as unhealthy behaviors [54], adverse emotions [55], and poorer psychosocial health [56] have been documented in left-behind children.

### Limitations

The current study has a few limitations to discuss. First, due to the study’s exploratory cross-sectional nature, it is difficult to conclude to what extent the earthquake had an effect on those who experienced it, as no pre-disaster or short-term after-disaster comparison data was available. Second, although the study was conducted adequately by trained investigators, data collection relied solely on the retrospective self-reports of the survivors 6 years after the Yushu earthquake, which can be subject to recall and desirability biases. Despite these limitations, the present study is one of only a few cross-sectional studies focusing on the long-term health status and associated risk factors in child survivors following an earthquake in rural China.

## Conclusions

In conclusion, the results of the present study help to expand our knowledge regarding the health status of child survivors 6 years after the Yushu earthquake. The results suggest the importance of greater vigilance and long-term attention to the children who lived in the rural plateau area and were most affected by the earthquake. This study provides important information regarding monitoring the long-term course of physical and mental health recovery of adolescents after earthquakes, and is of great significance for public awareness and the sensitivity towards those who were the most affected. Specific care, earthquake escape training, and long-term psychological interventions should be provided to adolescent survivors with relatively low health status, including those who were injured in the earthquake and those who were not living with parents.
